# Rhein Exhibits Anti-Inflammatory Effects in Chronic Atrophic Gastritis via Nrf2 and MAPK Signaling

**DOI:** 10.5152/tjg.2023.22251

**Published:** 2023-05-01

**Authors:** Song Liu, Lei Shu, Jiayao Yang, Yi Liu, Shu Zhang, Nian Wang, Zhaohong Shi

**Affiliations:** Department of Gastroenterology, Wuhan Hospital of Traditional Chinese and Western Medicine, Wuhan, Hubei, China

**Keywords:** Chronic atrophic gastritis, inflammation, MAPK signaling, Nrf2, rhein

## Abstract

**Background:**

Chronic atrophic gastritis is a premalignant lesion with a high risk of developing into gastric cancer. Rhein is a key active ingredient of several traditional Chinese medicines with multiple pharmacological effects. Nevertheless, the role of rhein in chronic atrophic gastritis is unclear.

**Methods:**

*Helicobacter pylori* infection was used to establish chronic atrophic gastritis in a mouse model. Murine gastric mucosa treated with saline or rhein was used in experiments. Hematoxylin and eosin staining and Alcian blue-periodic acid-Schiff staining were utilized for histopathological observation of murine gastric mucosa. The levels of proinflammatory factors were detected by enzyme-linked immunosorbent assay, and oxidative stress-associated markers were detected by commercially available assay kits. Western blotting was used for measuring the levels of nuclear factor, erythroid 2-like bZIP transcription factor 2, and mitogen-activated protein kinase signaling-related proteins.

**Results:**

Rhein mitigated the gastric mucosal injury and suppressed inflammation and oxidative stress in *H. pylori*-infected chronic atrophic gastritis mouse models. Rhein inactivated mitogen-activated protein kinase and activated erythroid 2-like bZIP transcription factor 2 signaling in gastric mucosa of mice with chronic atrophic gastritis.

**Conclusion:**

Rhein exhibits anti-inflammatory and antioxidant effects in chronic atrophic gastritis via erythroid 2-like bZIP transcription factor 2 and mitogen-activated protein kinase signaling.

**Main Points**

- Rhein mitigates *Helicobacter pylori*-induced gastric mucosal injury.
- Rhein restrains inflammation in gastric mucosa of chronic atrophic gastritis (CAG) mouse models.
- Rhein inhibits oxidative stress in gastric mucosa of mice with CAG.
- Rhein inactivates mitogen-activated protein kinase and activates erythroid 2-like bZIP transcription factor 2 signaling pathways.

## Introduction

Chronic atrophic gastritis (CAG) is a chronic digestive disorder characterized by atrophy of gastric mucosa epithelium and glands, reduced number of glands, thinning of gastric mucosa, thickening of mucosal base layer, or accompanied by pyloric and intestinal metaplasia.^1^ Most cases of CAG do not exhibit obvious clinical manifestations, and patients with CAG might suffer from gastric cavity fullness, pain, indigestion, belching, and anemia.^2^ Chronic atrophic gastritis is a disease with multiple pathogenic factors, including *Helicobacter pylori* (*H. pylori*) infection, poor dietary habits, environmental factors, and genetic factors.^3^ Among these factors, *H. pylori* infection is the main cause of CAG and other gastric disorders.^4^ *H. pylori* is a gram-negative, spiral bacterium that colonizes the human stomach.^5^ In view of this, *H. pylori* infection was used to establish the CAG in mouse model. Chronic atrophic gastritis is a premalignant lesion that is strongly associated with an elevated risk of gastric cancer.^6^ Hence, finding a novel effective approach for treating CAG is of great value.

Gastritis is defined as gastric mucosal inflammation.^7^ Mounting evidence has validated that sustained inflammatory response of gastric mucosa is the major inducement of CAG.^8,9^ Inflammation is characterized by increased levels of proinflammatory cytokines like interleukin-1 beta (IL-1β), IL-6, tumor necrosis factor-alpha (TNF-α), and cyclooxygenase-2 (COX-2).^10,11^ Prostaglandin E_2_ (PGE_2_) is a downstream product of COX-2 and also plays a significant role in the regulation of inflammation.^12^ Moreover, oxidative stress is considered to be a crucial factor in the pathogenesis of CAG.^13^ For example, fermented kimchi can ameliorate CAG by attenuating *H pylori*-related oxidative stress and endoplasmic reticulum stress.^14^ The major indicators of oxidative stress include oxidants like malondialdehyde (MDA) and antioxidants like superoxide dismutase (SOD).^15^

Rhein (4,5-dihydroxyanthraquinone-2-carboxylic acid) is the major component of several traditional Chinese medicines, including Sennae folium, rhubarb, and aloe, which have been indicated to alleviate edema, inflammation, and oxidative stress in multiple disorders.^16^ For example, rhein was reported to exert a protective effect on uric acid nephropathy by suppressing the inflammatory response.^17^ Additionally, rhein mitigates β-amyloid-induced oxidative stress in Alzheimer’s disease by promoting SIRT1/PGC-1α signaling activation.^18^ Rhein was also reported to be a critical ingredient of *San-Huang-Xie-Xin-Tang*, which was used for treating gastritis.^19^ The above evidence suggests that rhein might exert a gastroprotective impact on gastric injury. However, whether rhein has an effect on CAG is unclear. It was reported that rhein is able to inhibit lipopolysaccharide (LPS)-induced inflammation and oxidative stress in an intestinal barrier injury rat model via nuclear factor, erythroid 2 (NFE2)-like bZIP transcription factor 2 (Nrf2, also known as NFE2L2), and mitogen-activated protein kinase (MAPK) pathways.^20^ Furthermore, activation of Nrf2 and MAPK signaling has been demonstrated to be involved in affecting gastric injury.^21,22^

This study aimed to probe the function and potential mechanism of rhein in *H. pylori*-infected CAG mouse models. It was hypothesized that rhein might have an effect on inflammation and oxidative stress in CAG by regulating Nrf2 and MAPK pathways. Our findings might provide a new option for treating CAG.

## Materials and Methods

### H. pylori Culture

*H. pylori* strain ATCC43504 was obtained from American Type Culture Collection (Manassas, Va, USA) and cultured as previously described.^23^ The bacteria were incubated on trypticase soy (TS) agar plates supplemented with 5% sheep blood at 37°C for 3 days under microaerophilic conditions (CampyGen Atmosphere Generation Systems, Thermo Scientific, Vienna, Austria). Then, the bacteria were collected in clean TS broth, subjected to centrifugation at 3000 g for 5 minutes, and resuspended in the broth at 10^9^ colony-forming units (CFUs)/mL. Cultures incubated for 72 hours on plates were used in the following experiments.

## Animal Models and Drug Treatment

**Figure 1.**
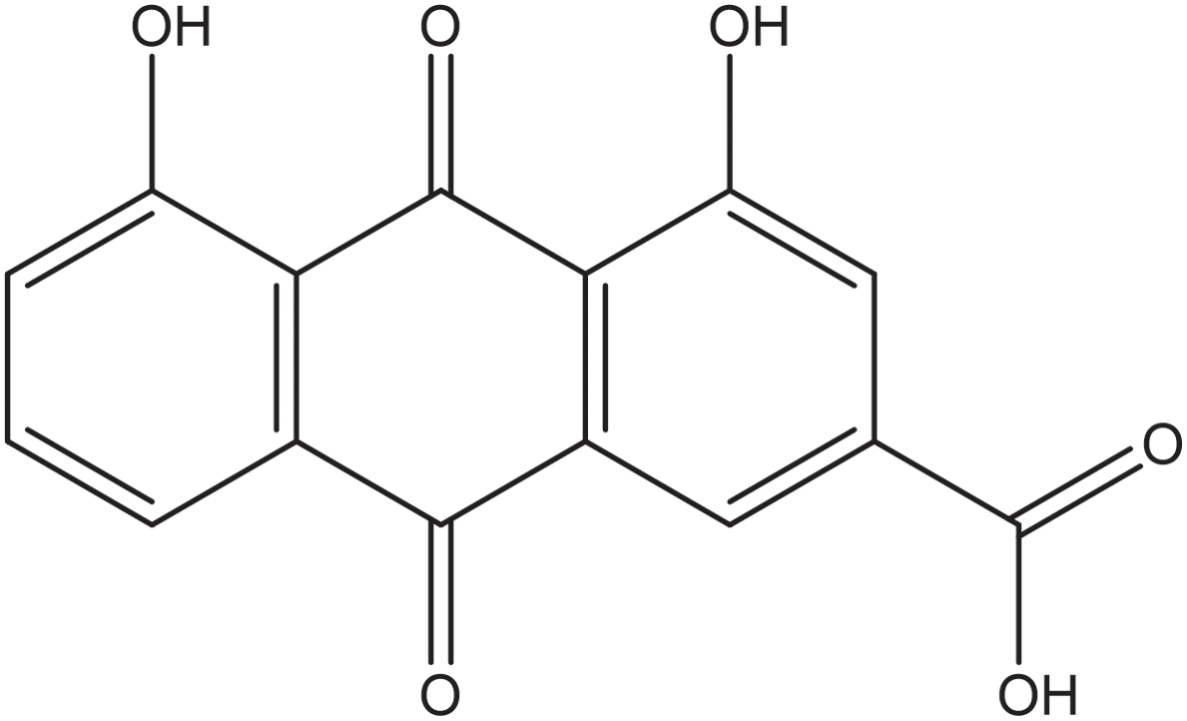
Chemical structure of rhein.

Totally 40 male C57BL/6 mice (5-week-old) were purchased from HFK Bioscience (Beijing, China). The CAG mouse model was established according to previous studies.^1,23^ The mice were randomly divided into 4 groups: sham + normal saline (NS), sham + rhein, CAG + NS, and CAG + rhein (n = 10 per group). To establish the CAG mouse model, mice in the CAG groups were treated with *H. pylori* infection combined with high-salt diet. In brief, the mice were injected intraperitoneally with pantoprazole (20 mg/kg, 3 times/week; Pacific Pharma, Korea) as proton pump inhibitor to enhance the successful colonization of* H. pylori* by reducing gastric acid. Afterward, gastric intubation needles were used to inoculate the stomach of the mice with 10^8^ CFUs/mL *H. pylori* suspension or an equal volume (0.1 mL) of clean TS broth (4 times/week, for 24 weeks). In parallel, the mice were fed with high-salt diet containing 8% NaCl (AIN-76A; Biogenomics, Seongnam, Korea). Mice in the sham groups received the same procedure without *H. pylori* infection and high-salt diet. Rhein (purity ≥ 98%) was purchased from Sigma-Aldrich (St. Louis, Mo, USA), and its chemical structure was shown in Figure 1. Twenty-four weeks later, mice in the sham + rhein and the CAG + rhein groups were orally administrated with 100 mg/kg rhein (once daily, for 8 weeks)^16,24^ and mice in the sham + NS and the CAG + NS groups received saline treatment. All animal experiments were approved by the Animal Ethics Committee of Wuhan Myhalic Biotechnology Co., Ltd (No: 20210617088) and complied with the guidelines of the National Institutes of Health Guide for the Care and Use of Laboratory Animals.

### Sample Collection

The drug was stopped after 8 weeks, and mice were fasted for 24 hours before the end of the experiment. Mice were anesthetized with sodium pentobarbital (50 mg/kg) by intraperitoneal injection. Blood samples were collected from the abdominal aorta with a syringe, injected into the blood collection tube for coagulation, and centrifuged at 3000 rpm for 15 minutes. Then, the serum was harvested and stored at –70°. Mice were then sacrificed by cervical dislocation under anesthesia, and murine gastric mucosa was collected. A portion of gastric mucosa was fixed with formalin for histological analysis and the remaining was stored at –80° for further experiments.

### Hematoxylin and Eosin Staining

Murine gastric mucosa was fixed with 10% formalin and embedded in paraffin. Tissue sections of 4 µm thickness were dewaxed with xylene solution, hydrated with ethanol gradient, and subsequently stained with hematoxylin and eosin solution (Sigma-Aldrich). After being washed with 95% ethanol for 1 minute and xylene for 3 times, the slices were sealed with neutral gum and a microscope (Nikon, Tokyo, Japan) was used for histopathological observation. The inflammation score was used to show the level of inflammatory cell infiltration from 0 (none) to 3 (all mucosa). The erosion score was defined as proportion of erosive lesions from 0 (none) to 3 (all mucosa). Pathological score of hematoxylin and eosin (HE) was computed as erosion sore + inflammation score.^23^

### Alcian Blue-Periodic Acid-Schiff) Staining

Murine gastric tissue sections were stained with Alcian blue (Sigma-Aldrich) dropwise for 10 minutes, treated with 0.5% periodic acid solution, and washed in running water. After being washed twice in distilled water, tissue sections were drip-stained with Schiff reagent (Sigma-Aldrich) for 20 minutes away from light. Then, the sections were rinsed and dried. After that, Mayer hematoxylin was used to stain the nucleus lightly for 1 minute. Eventually, the slices were rinsed, dehydrated, sealed with neutral gum, and observed under a microscope (Nikon) for analyzing mucous metaplasia.

### Enzyme-Linked Immunosorbent Assay

Concentrations of proinflammatory factors (TNF-α, COX-2, IL-6, and IL-1β) in gastric mucosa and PGE_2_ in murine serum were detected by the following mouse enzyme-linked immunosorbent assay kits: TNF-α (ab100747; Abcam, Cambridge, UK), COX-2 (SEKM-0156; Solarbio, Beijing, China), IL-6 (BMS603-2; Invitrogen, Carlsbad, Calif, USA), IL-1β (ab197742, Abcam), PGE_2_ (SEKM-0173, Solarbio), respectively, according to the instructions of manufacturers.

### Measurement of Oxidative Stress-Related Markers

Levels of oxidative stress-associated markers were measured by commercially available assay kits. Myeloperoxidase (MPO) activity and contents of MDA and SOD were determined by MPO assay kit (A044-1-1, colorimetric method), MDA assay kit (A003-1-1, TBA method), and SOD assay kit (A001-3-2, WST-1 method) (all from Nanjing Jiancheng Bioengineering Institute, Nanjing, China), respectively, following the manufacturer’s protocols.

### Western Blotting

Proteins were isolated from murine gastric mucosa using Radio Immunoprecipitation Assay (RIPA) lysis buffer (Beyotime, Shanghai, China) supplemented with 1 mM phenylmethylsulfonyl fluoride (Sigma-Aldrich). After incubation for 30 minutes and centrifugation at 12 000 g at 4°C for 15 minutes, the supernatants were harvested, and the proteins were quantified with a bicinchoninic acid (BCA) assay kit (Beyotime). Subsequently, protein samples (30 µg) were separated by 10% sodium dodecyl sulfate polyacrylamide gel electrophoresis (SDS-PAGE) gels, transferred to polyvinylidene fluoride membranes (Invitrogen), which were then blocked with 5% defatted milk, and incubated at 4°C overnight with the primary antibodies as follows: anti-Nrf2 (ab92946, 1:1000), anti-Heme oxygenase 1 (HO-1) (ab52947, 1:2000), anti-p-Jun N-terminal kinase (JNK)1/2 (ab124956, 1:1000), anti-JNK1/2 (ab179461, 1:1000), anti-p-p38 (ab195049, 1:1000), anti-p38 (ab170099, 1:1000), and anti-β-actin (ab8227, 1:1000) (all from Abcam). Afterward, the membranes were incubated with the secondary antibody (ab288151, Abcam). Eventually, protein bands were visualized with an enhanced chemiluminescence (ECL) detection kit (Cwbiotech, Beijing, China) and quantified with Image Lab 3.0 software (Bio-Rad, Hercules, Calif, USA).

### Statistical Analysis

Statistical analysis was performed using Statistical Package for the Social Sciences 16.0 software (SPSS Inc.; Chicago, IL, USA). Data are presented as the mean ± standard devi­ation. Student’s *t*-test was utilized for 2 group comparisons, while analysis of variance followed by Tukey’s post hoc analysis was used for multiple group comparisons. The value of *P* < .05 was regarded as statistically significant.

## Results

### Rhein Mitigates Gastric Mucosal Injury

**Figure 2.**
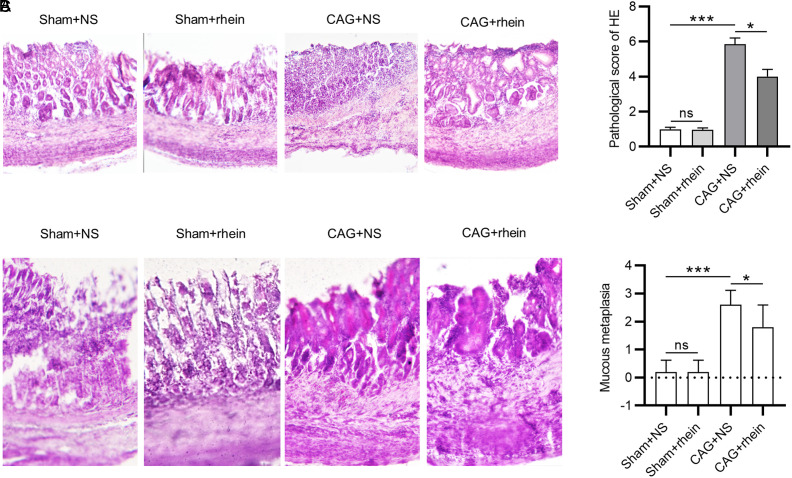
Rhein mitigates gastric mucosal injury. (A-B). HE staining for evaluating the histopathological changes of murine gastric mucosa in each group. (C-D). AB-PAS staining for assessing the changes in mucous metaplasia of murine gastric mucosa. ^*^P < .05, ^***^P < .001. HE, hematoxylin and eosin; AB-PAS, Alcian blue-periodic acid-Schiff.

We first examined the effect of rhein on the pathology of gastric mucosa of mice with CAG. As shown by HE staining, compared with the sham group, mice in the CAG + NS group exhibited a disordered structure of gastric mucosal epithelium, a reduction in glands and sparse arrangement, accompanied by inflammatory cell infiltration (Figure 2A). Intriguingly, it was displayed that the pathological conditions of gastric mucosa were improved after rhein treatment (Figure 2A and 2B). A similar trend was observed in the results of Alcian blue-periodic acid-Schiff staining. The CAG + NS group displayed evident mucous metaplasia relative to the sham group, which was ameliorated in the rhein-treated CAG mice (Figure 2C and 2D). These suggest that rhein can mitigate *H. pylori*-induced gastric mucosal injury in mice with CAG.

### Rhein Restrains the Inflammation in Murine Gastric Mucosa

**Figure 3.**
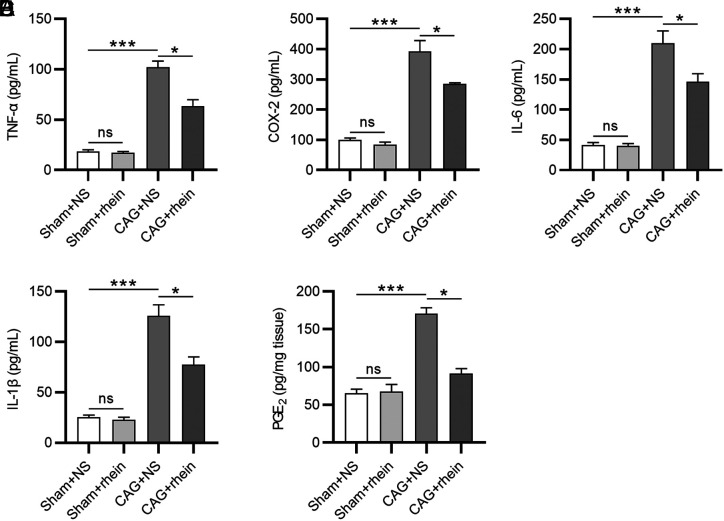
Rhein restrains the inflammatory response in murine gastric mucosa. (A-E). ELISA for assessing concentrations of proinflammatory cytokines (TNF-α, COX-2, IL-6, and IL-1β) in murine gastric mucosa and PGE_2_ in murine serum. ^*^P < .05, ^***^P < .001. ELISA, enzyme-linked immunosorbent assay; IL, interleukin; TNF- α, tumor necrosis factor-alpha; COX-2, cyclooxygenase-2; PGE_2, _prostaglandin E_2_.

Next, we tested whether rhein influenced the inflammatory response in CAG. Enzyme-linked immunosorbent assay was used to assess the levels of proinflammatory cytokines. As revealed by the results, high levels of proinflammatory cytokines (TNF-α, COX-2, IL-6, and IL-1β) were shown in the CAG + NS group, while rhein treatment led to a decrease in the cytokine levels (Figure 3A-D). Then, we evaluated the production of serum PGE_2_, a downstream product of COX-2. In comparison to the sham group, the level of serum PGE_2_ was evidently higher in the CAG + NS group and rhein treatment was shown to reduce its level (Figure 3E), indicating that rhein greatly suppresses the inflammation in gastric mucosa infected by *H. pylori.*

### Rhein Inhibits Oxidative Stress in the Gastric Mucosa

**Figure 4.**
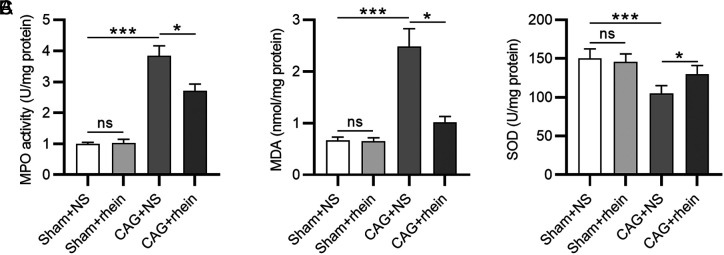
Rhein inhibits oxidative stress in the gastric mucosa. (A-C). Measurement of MPO, MDA, and SOD levels in murine gastric mucosa. ^*^P < .05, ^***^P < .001. MPO, myeloperoxidase; MDA, malondialdehyde; SOD, superoxide dismutase.

We subsequently explored the function of rhein in regulating oxidative stress in CAG. Myeloperoxidase is a peroxidase enzyme that catalyzes the release of cytotoxic hypochlorite and other chlorinated species, resulting in potential oxidative stress.^25^ As a result, MPO activity was evaluated by colorimetric method, which showed that MPO activity was remarkedly increased in CAG group compared to that in the sham group (Figure 4A). However, the increased MPO activity was reduced by treatment of rhein (Figure 4A). After that, we assessed the levels of oxidant MDA and antioxidant SOD. Notably, the gastric mucosa of mice with CAG showed elevated production of MDA and decreased production of SOD compared with those of the normal mice (Figure 4B and 4C). The altered levels of MDA and SOD were both reversed after rhein treatment, suggesting that rhein can mitigate *H. pylori*-induced oxidative stress in mice with CAG.

### Rhein Activates Erythroid 2-Like bZIP Transcription Factor 2 and Inactivates Mitogen-Activated Protein Kinase Signaling Pathways

**Figure 5.**
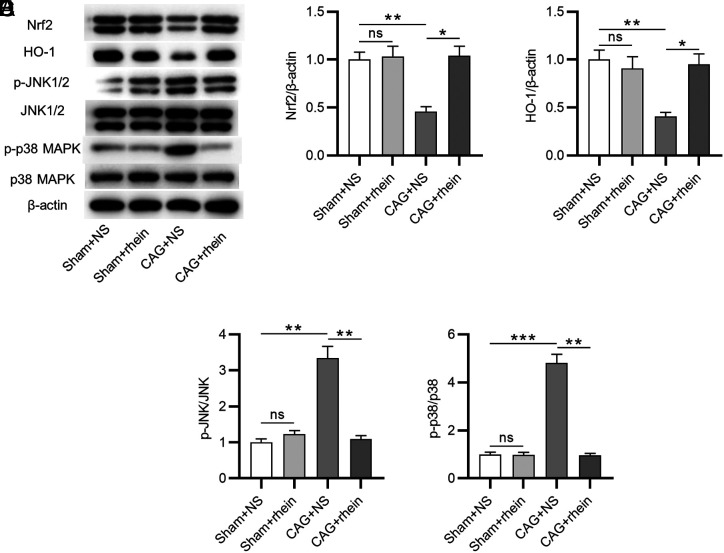
Rhein inactivates MAPK signaling and activates Nrf2 signaling. (A-E). Western blotting for evaluating the levels of Nrf2 and MAPK signaling-associated proteins in murine gastric mucosa. ^*^P < .05, ^**^P < .01, ^***^P < .001. MAPK, mitogen-activated protein kinase; Nrf2, erythroid 2-like bZIP transcription factor 2.

To reveal the potential mechanism of rhein underlying CAG, we examined whether rhein had an effect on Nrf2 and MAPK pathways. Western blotting was utilized to evaluate the levels of Nrf2 and MAPK signaling-associated proteins (Figure 5A). As displayed by the results, Nrf2 and HO-1 protein levels were markedly reduced in CAG + NS group (Figure 5B and 5C). However, the effects were shown to be partially reversed after rhein treatment (Figure 5B and 5C), suggesting that rhein activates Nrf2 signaling in CAG. Next, we analyzed the protein levels of MAPK family members JNK and p38. Compared with the sham group, the ratios of phosphorylated (p)-JNK1/2 to total JNK1/2 and p-p38 to total p38 in the CAG + NS group were all increased; however, the increased levels were markedly reduced after treatment of rhein (Figure 5D and 5E). This indicates that pretreatment of rhein in murine gastric mucosa can inactivate MAPK signaling.

## Discussion

Chronic atrophic gastritis is a precancerous lesion that has a high risk of developing into gastric cancer.^26^ *H. pylori* infection is regarded as the major inducement of CAG.^27^ Previous studies have demonstrated that *H. pylori* infection induces oxidative stress and inflammation in gastric mucosa, consequently resulting in adverse gastric mucosal injury.^14,28^ Thus, we established *H. pylori*-infected mouse models of CAG in this study. Rhein, a natural polymer with high safety, has proven anti-inflammatory, anti-edema, and antibacterial capabilities.^29^ Owing to its wide properties, rhein has been used in multiple diseases. For example, rhein attenuates LPS-induced injury by inhibiting the inflammatory response in intestinal epithelial cells.^30^ Importantly, rhein was reported to suppress ethanol-induced inflammation and oxidative stress in gastric ulcer, exhibiting a gastroprotective effect.^13^ In the present study, rhein was shown to improve the disordered structure and mucous metaplasia of gastric mucosa in CAG mice induced by *H. pylori*, confirming that rhein exerts a protective role in CAG.

Inflammation is featured with enhanced production of proinflammatory factors including TNF-α, COX-2, IL-6, and IL-1β.^31^ Additionally, COX-2 participates in the conversion of arachidonic acid into PGH_2_, a precursor of PGE_2_ which further promotes the inflammatory response in tissues.^32^ In accordance with previous evidence, it was found in this study that rhein treatment reversed the elevated secretion of proinflammatory factors in gastric mucosa of mice with CAG, indicating that rhein has an anti-inflammatory effect on *H. pylori*-induced inflammation in mice with CAG. Furthermore, MPO has been indicated to mediate oxidative stress and function as a biomarker of neutrophil infiltration.^33^ Neutrophil infiltration plays a pivotal role in the development of oxidative stress-induced inflammatory response.^34^ Overproduction of MPO and its mediated oxidative stress can contribute to inflammation and lead to severe tissue damage.^25^ In this study, MPO activity in gastric mucosa of mice with CAG was significantly high but was decreased upon rhein treatment. An imbalance between the production of oxidants and antioxidants results in oxidative stress, which exacerbates the condition of CAG.^35^ In the current study, rhein-treated CAG mice displayed a decreased level of the oxidant MDA and an increased level of the antioxidant SOD compared to those of NS-treated mice. Collectively, the above results demonstrate that rhein can alleviate the oxidative stress in gastric mucosa of mice with CAG.

Emerging evidence has indicated that inflammation and oxidative stress in gastric injury are mediated by various signaling pathways.^36,37^ A previous study showed that rhein plays anti-inflammatory and antioxidant roles in intestinal injury via Nrf2 and MAPK pathways.^20^ Erythroid 2-like bZIP transcription factor 2 is known as transcription factor that exerts a significant effect on cellular defense against *H. pylori* infection-induced injury including oxidative stress.^23^ Upon oxidative stress, Nrf2 can bind to antioxidant response element and regulate the expression of antioxidant genes like HO-1.^20^ Moreover, previous studies have demonstrated that the Nrf2/HO-1 signaling pathway-related oxidative stress is involved in inflammation and apoptosis of cells.^38,39^ Here, we found that *H. pylori* induced the downregulation of Nrf2 and HO-1 proteins in murine gastric mucosa. Nevertheless, treatment of rhein effectively restrained the above effects caused by *H. pylori*. Furthermore, the impact of rhein on MAPK signaling pathway was also tested. Anti-p-Jun N-terminal kinase and p38 MAPK are key members of the MAPK family which have been indicated to modulate diverse cellular activities, including inflammatory response.^40^ Results from this study revealed that rhein reversed *H. pylori* infection-induced phosphorylation of JNK and p38 in gastric mucosal tissues of mice with CAG. Hence, the above results indicated that rhein can inactivate MAPK signaling and activate Nrf2 signaling in murine gastric mucosa.

However, there are some limitations to this study. The study included a limited number of animals. Future studies with larger sample size and prospective trials are needed. Additionally, more signaling pathways that might be mediated by rhein in CAG need to be further investigated in future studies.

In conclusion, we explored the function and potential mechanism of rhein in regulating inflammation and oxidative stress in a CAG mouse model. The results reveal that rhein can mitigate gastric mucosal injury and suppress inflammation and oxidative stress in murine gastric mucosa by activating Nrf2 and inactivating MAPK signaling pathways. Our findings might provide a new option for CAG therapy. Translational medicine research or clinical trials upon rhein are needed in the future.
